# Development and Implementation of a Web-Enabled 3D Consultation Tool for Breast Augmentation Surgery Based on 3D-Image Reconstruction of 2D Pictures

**DOI:** 10.2196/jmir.1903

**Published:** 2012-02-03

**Authors:** Pablo de Heras Ciechomski, Mihai Constantinescu, Jaime Garcia, Radu Olariu, Irving Dindoyal, Serge Le Huu, Mauricio Reyes

**Affiliations:** ^1^Institute for Surgical Technology and BiomechanicsUniversity of BernBernSwitzerland; ^2^Department of Plastic, Reconstructive, and Aesthetic SurgeryUniversity Hospital, InselspitalUniversity of BernBernSwitzerland; ^3^Crisalix SALausanneSwitzerland; ^4^La ClinicMontreuxSwitzerland

**Keywords:** Medical informatics computing, computer-assisted surgery, imaging, three-dimensional

## Abstract

**Background:**

Producing a rich, personalized Web-based consultation tool for plastic surgeons and patients is challenging.

**Objective:**

(1) To develop a computer tool that allows individual reconstruction and simulation of 3-dimensional (3D) soft tissue from ordinary digital photos of breasts, (2) to implement a Web-based, worldwide-accessible preoperative surgical planning platform for plastic surgeons, and (3) to validate this tool through a quality control analysis by comparing 3D laser scans of the patients with the 3D reconstructions with this tool from original 2-dimensional (2D) pictures of the same patients.

**Methods:**

The proposed system uses well-established 2D digital photos for reconstruction into a 3D torso, which is then available to the user for interactive planning. The simulation is performed on dedicated servers, accessible via Internet. It allows the surgeon, together with the patient, to previsualize the impact of the proposed breast augmentation directly during the consultation before a surgery is decided upon. We retrospectively conduced a quality control assessment of available anonymized pre- and postoperative 2D digital photographs of patients undergoing breast augmentation procedures. The method presented above was used to reconstruct 3D pictures from 2D digital pictures. We used a laser scanner capable of generating a highly accurate surface model of the patient’s anatomy to acquire ground truth data. The quality of the computed 3D reconstructions was compared with the ground truth data used to perform both qualitative and quantitative evaluations.

**Results:**

We evaluated the system on 11 clinical cases for surface reconstructions and 4 clinical cases of postoperative simulations, using laser surface scan technologies showing a mean reconstruction error between 2 and 4 mm and a maximum outlier error of 16 mm. Qualitative and quantitative analyses from plastic surgeons demonstrate the potential of these new emerging technologies.

**Conclusions:**

We tested our tool for 3D, Web-based, patient-specific consultation in the clinical scenario of breast augmentation. This example shows that the current state of development allows for creation of responsive and effective Web-based, 3D medical tools, even with highly complex and time-consuming computation, by off-loading them to a dedicated high-performance data center. The efficient combination of advanced technologies, based on analysis and understanding of human anatomy and physiology, will allow the development of further Web-based reconstruction and predictive interfaces at different scales of the human body. The consultation tool presented herein exemplifies the potential of combining advancements in the core areas of computer science and biomedical engineering with the evolving areas of Web technologies. We are confident that future developments based on a multidisciplinary approach will further pave the way toward personalized Web-enabled medicine.

## Introduction

Since the creation of the World Wide Web in the early 1990s, its use for medical applications has attracted much attention due to the possibilities of centralized storage and the efficient sharing of information. The creation of the picture archiving and communication system and related Web-enabled interfaces for the Internet demonstrates the interest from the medical community in accessing information in a reliable, economical, and convenient way [1,2]. However, despite efforts in computer sciences (eg [3,4]), the processing of medical images is still computationally expensive for real-time use on most personal computers. Continuing efforts are being made toward personalized patient models for the predictive health care of the future [5], leading to new pathways in health care [6].

A field in which Internet capabilities can be used for medical purposes is 3-dimensional (3D) human anatomy. Contrary to Web-enabled medical tools for education purposes, where standard data models are employed, the scenario is more complex when considering confidential patient-specific or personalized medical imaging data from 2-dimensional (2D) pictures. Therefore, the tool presented in this paper was developed and tested in a multidisciplinary effort by a team of experts consisting of surgeons, biomedical engineers, computer graphic specialists, and Web developers and designers.

In breast augmentation surgery, surgeon–patient communication is vital, as the diagnosis, treatment, and outcome are dominated by the patient’s subjective assessment of the visual results of the elective surgical procedure. Failure to meet the patient’s expectations (augmentation volume, breast projection, etc) can lead to the need for reoperations and ultimately to legal action. It is therefore essential that patients be personally involved in the process of implant selection, supported by a realistic visual representation of their body, the previsualization of the final result. The success of the surgical outcome depends significantly on the choice of implant shape, size, projection, and anatomical placement, and these are key factors in the decision process.

Available computerized 3D anatomical visualization can be divided into the following categories. First, image-morphing techniques are software solutions working exclusively in 2 dimensions, where a patient’s photograph might or might not be the basis for the projected postoperative result (eg, PhotoShop, ReShapr [7], PlasticSurgerySimulator [8]). Second, templated and predefined software allows a user to define a set of parameters and to relate them to a predefined model and a predefined outcome (eg, BreastDoctors [9], LoveYourLook [10]). Third, educational software has been one of the areas where 3D Web-based medicine has shown success. For instance, the use of avatars in virtual reality learning environments [11-13] has captured the attention of researchers and medical practitioners due to the dynamic and engaging learning, peer collaboration, and interaction with users around the world [14-18]. However, these do not meet the requirements of individualized patient data analysis. Fourth, 3D scans allow an accurate 3D reconstruction of the patient’s specific shape, texture, color, and sizing. Most scanning hardware such as Portrait3D (www.axisthree.com) and VECTRA 3D (www.canfieldsci.com) requires bulky and costly equipment that must be installed at each surgeon’s office.

The above-mentioned techniques have inherent limitations for application in the daily clinical work of a surgeon.

We propose a patient-specific system for breast augmentation previsualization using well-established 2D digital photos of the patient’s body taken with a digital camera (together with a few extra body measurements for scaling) and transforming them into a 3D, interactive, visual surface representation of the upper torso, which is then available for interactive planning to the user. The simulation is performed on dedicated servers, accessible via Internet. It allows the surgeon, together with the patient, to previsualize the impact of the proposed breast augmentation directly during the consultation before a surgery is decided upon. The hypothesis of this study was thus that the proposed Web-based system would allow previsualization of results, with varying implant size and varying implant locations, acting as a guide for the preoperative planning and decision process.

The 3 goals of this study were thus to (1) develop a computer tool that allows the individual reconstruction and simulation of 3D soft tissue from ordinary digital photos of breasts, (2) implement a Web-based, worldwide-accessible preoperative surgical planning platform for plastic surgeons, and (3) validate this tool through a quality control analysis by comparing 3D laser scans of the patients with the 3D reconstructions made using this tool from original 2D pictures of the same patients.

## Methods

The following subsections describe some particular adopted strategies, giving particular emphasis to Web-related components and data management.

**Figure 1:**
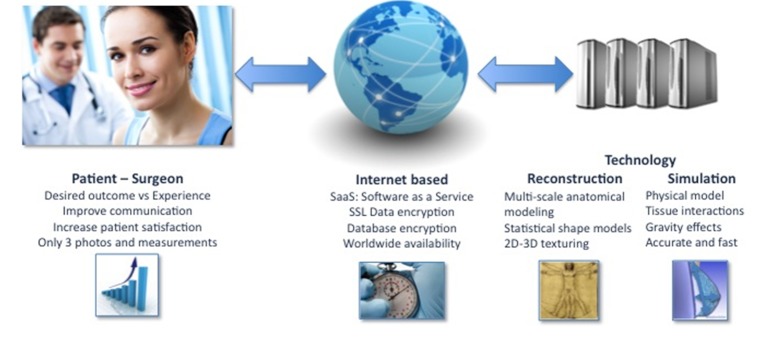
General overview of the developed system. An Internet-based solution combining advanced technologies enables a realistic, patient-specific, simulated clinical scenario. 2D = 2-dimensional; 3D = 3-dimensional.

Figure 1 illustrates the general pipeline of the developed system. Patients and clinicians are connected through the Internet, enabling a dialogue while being independent of geographical location. Personalized patient information, such as digital photographs and sparse measurements, enable the modeling and simulation framework to create a completely patient-specific clinical scenario.

### Retrieval and Analysis of Patient-Specific Information

The real-time generation of a 3D model of the patient’s anatomy is based on the extraction of patient-specific information, provided by the user in the form of 2D digital pictures, taken at 3 different angles (frontal and lateral images). In addition, and in order to create a plausible model, 2 physical distance measurements of the patient’s anatomy are requested, such as nipple-to-nipple and nipple-to-submammary fold. The set of 2D images and sparse measurements allow for calibration of images to the actual patient’s anatomy and for reconstruction of a realistic model of the patient’s anatomy.

An important aspect is to provide the user with understandable information regarding the way digital pictures need to be taken. This is indicated to the user as guidelines for taking suitable patient photos; generally, plain white hospital walls offer sufficient contrast. Conventional fluorescent lighting found in offices and hospitals is perfectly acceptable for the system to be able to reliably detect the patient’s contour from the 2D pictures.

**Figure 2:**
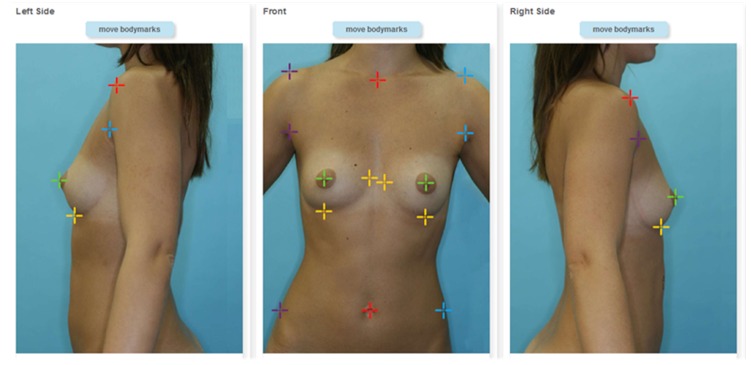
Three landmarked photos of a patient. Visual aids on where to place landmarks and a simple Web interface guide the user through the annotation of images. Cropped screenshot taken from the Web-based interface.

To initiate the body extraction algorithm, the user is required to define a few anatomical landmarks on each of the 3 pictures (see Figure 2). This step is guided interactively on the website and takes less than 5 minutes. From the result of the body extraction algorithm, several curvature characteristics of the breast are computed, to automatically determine the patient’s breast type and to fine-tune the 3D reconstruction algorithm.

### 3D Reconstruction of a Patient’s Anatomy and Web 3D Visualization

**Figure 3:**
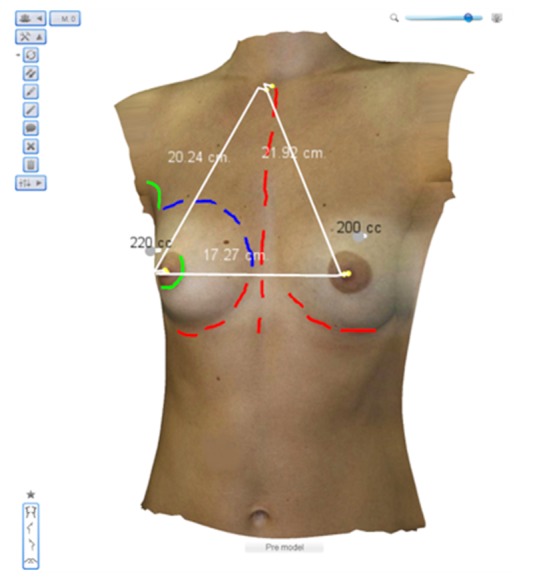
Web-based 3-dimensional (3D) annotations on a reconstructed patient model. A set of tools including 3D distances, text, and body drawing enable a personalized virtual clinical analysis. Cropped screenshot taken from the Web-based interface.

Once body extraction and breast type characterization is finished, a specialized image-based 3D/2D reconstruction algorithm is used to estimate the 3D shape of the patient’s anatomy, from the imaging and morphometric information provided by the user. The user is presented with a 3D visual representation of the 3 views, in the form of a textured surface model (see Figure 3). The model is interactive and can be rotated by the user in the Web browser. Two technologies seem to be taking the lead in Web3D: WebGL (http://www.khronos.org/webgl/) and Unity3D (http://unity3d.com/). The first is a continuation of the O3D project from Google; despite its initial progress the engine has lost some impact due to browser incompatibilities (especially Internet Explorer) and security-related concerns raised by the community. Unity3D is a commercial product with a growing and active community, available on multiple platforms. The drawback of Unity3D is the plug-in architecture, which might hinder its dissemination to less-experienced Web users.

3D annotations, such as floating text, lines, and landmarks, can be added directly to the models (Figure 3). In addition, 3D measurements can be performed on the digital model of the patient, as opposed to the traditional way of painting directly on the patient’s skin. Besides the less-invasive communication approach with the patient, the clinician has the advantage of storing and accessing the 3D model on demand and without the need for the patient to be present.

### Web-Enabled Biomechanical Simulations

A widely accepted breast augmentation procedure consists of choosing between three different implant placement techniques, such as subglandular, submuscular, and dual plane. The implants themselves come in a plethora of different widths, heights or projections, lengths, and shapes. It is in this large array of choices that use of a physics-based implant simulator on the virtual patient is important to quickly and decisively give an idea of a final postoperative result. Furthermore, interpatient anatomical variability adds to the complex decision-making process.

Consequently, a Web-enabled simulator for breast augmentation needs to consider the current breast augmentation techniques while adapting them to the Web. The following subsections introduce the Web-based planning and biomechanical simulator.

### Planning

**Figure 4:**
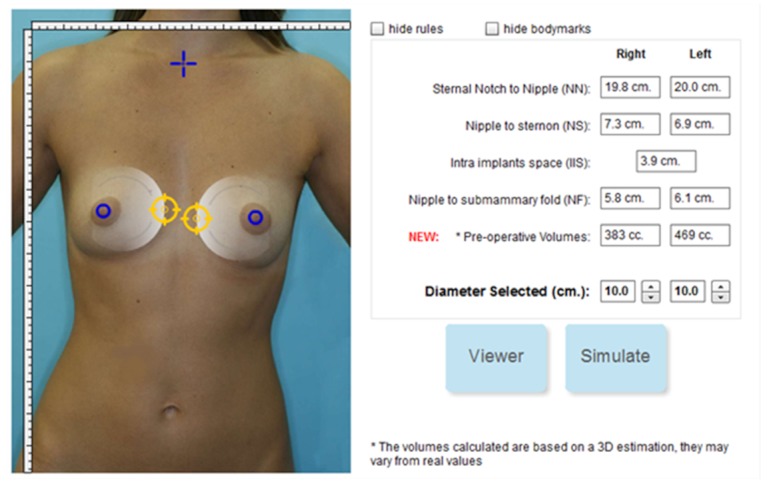
Selection of implant position and diameter. Cropped screenshot taken from the Web-based interface.

In the planning process, implant positions and diameters are defined for use during the simulation. Positioning and sizing can be indicated directly on the photo or defined numerically (Figure 4). The surgeon can also choose the implant brand from an implant catalogue, which is a database including various brands on the market. The implant is classified according to user requirements for diameter, projection, and volume. The three methods of implantation, subglandular, submuscular, and dual plane, are specified during this step.

### Biomechanical Simulator

To stay true to reality, many pathways have been explored in terms of viable simulation solutions such as fluidics [19], complex deformable models [20], pressure models [21], uniform mass-spring models [22], and mass tensor models [23]. First, the most important aspect of a simulation is robustness, such that a surgeon is confident it will enhance a consultation with a lifelike and consistent result. This is a challenging problem, since many parameters influence the final look, such as the shape and volume of the breasts and implants, gravity, skin tension, and the interactions between the implants and the internal tissues such as muscles, fat, glands, and skin. Second, the simulator must yield results in a reasonable time frame such that the surgeon and patient perceive the different choices in a noninterruptive manner. Third, the 3D graphical appeal of the visualization must be such that the skin, texture, and lighting conditions of the virtual reconstructed patient are smooth, so as to add to the realism of the model and emphasize the improvements to the patient’s appearance.

Instead of creating a simulator that builds from prefabricated examples, our platform is based on the physical properties of human tissue using the tissue elastic model (TEM), which closely resembles the finite element method. The most important features differentiating the two methods are that in TEM

- Deformations such as torsion, volume, and angular constraints are more relaxed.
- Speed of execution is emphasized, such that simulations of thousands of iterations on large and complex aggregates of voxelized tissue (defined below) are handled in a few seconds.
- A domain-specific implementation involving a biomechanics model focuses solely on the breasts.
- Tissue elasticity is inherent in the model.

The TEM engine has an elasticity module included that takes into account the existing degree of skin elasticity and type, which is chosen by the surgeon who submits the pictures. We took into consideration 4 elasticity types: loose, moderate, tight, and very tight. Based on the selected skin elasticity the biomechanical engine modulates the global outcome of the simulation. Systems with a similar basis of gridlike structures can be found [24,25] and differ from fluid dynamics [26,27] in that neighboring relations stay the same until the final simulation step, independent of deformations.

### Voxelization

**Figure 5:**
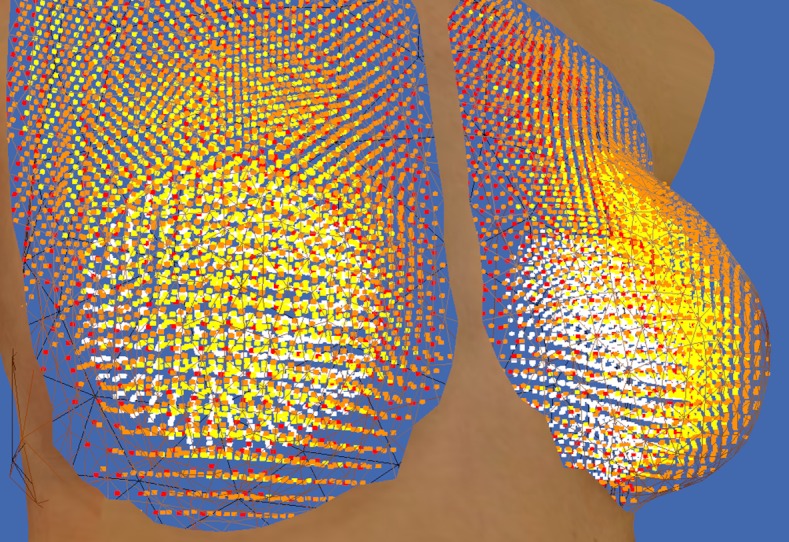
Voxelized breasts and implants. The fat layer is seen in yellow, the skin in orange, and the muscle layer in red. The implants are shown as white voxels or particles. This screenshot is taken from the simulation developer’s point of view and is not visible in the Web-based interface.

The simulator starts by defining the volume constraints of the breasts and by subdividing the breast tissue into tiny 3D cubes called voxels (or volumetric pixels). This process is called voxelization and volumetrically approximates the different tissue layers of the breasts such as muscles, fat, glands, and skin. The innermost layers are the torso and bones, followed by the muscle layer of the pectorals, then fat and glands, and finally skin. This biomechanical model is medically relevant to be faithful to the surgeon’s expected outcome (see Figure 5).

The simulator requires that the surgeon place the 2 implants in an image of the front part of the patient with the center part of the implant as visual guidance (see Figure 4). Once the placement is complete and the surgeon starts the simulation, the process starts by finding the space needed by the implant in the virtual patient’s tissue. Depending on whether the operation is subglandular, submuscular, or dual plane, the implant is placed between the rib cage and muscle (submuscular) or between muscle and glandules, (subglandular or dual plane).

### Quality Control Assessment of the System’s Precision

We retrospectively conducted a quality control assessment on available anonymized pre- and postoperative 2D digital photographs of patients undergoing breast augmentation procedures. The above-presented method was used to reconstruct 3D pictures from 2D digital pictures. A laser scanner (EScan3D [28]) capable of generating a highly accurate surface model of the patient’s anatomy was used to acquire ground truth data. We compared the quality of the computed 3D reconstructions against the ground truth data used to perform both qualitative and quantitative evaluations.

For a qualitative evaluation, an overlay of the reconstruction is superimposed on the laser scan and presented to 4 plastic surgeons for direct visual comparison. The scanner is capable of submillimeter precision and can capture the breast field in 3 overlapping sweeps, each sweep lasting between 5 and 10 seconds. Slight patient motion during laser acquisition of the surfaces introduces errors in the evaluation. The patient was required to hold her breath to minimize chest motion. Eder et al [29] reviewed techniques for scanning for breast surgery and discuss potential patient motion-related problems. Although small patient motion-induced errors are difficult to quantify accurately, they are nonetheless negligible with respect to the assumptions of the reconstruction from limited views.

Laser technology relies on several acquisitions, since the field of view is insufficient to cover the entire thorax. The patient is likely to move between acquisitions. To account for potential patient motion between scans, the patient is repositioned to stand with her back against the wall aligned with a patient-specific template with the head tilted back, and the elbow and scapula in contact with the wall. Testing of this protocol on several patients found that patient motion due to breathing was minimized, thus ensuring a good laser reconstruction. The alignment of the arms against the template also enables breast deformations to be easily matched during reconstruction of the individual 3D surface scans. These findings are also in agreement with Eder et al [29], who described virtual 3D modeling for breast surgery. Since the patient is standing with her back and elbows against the wall, she is able to relax her posture, and a better mediolateral positioning of the patient can be obtained. This is probably because the patient can rely on a spatial reference to set her posture.

**Figure 6:**
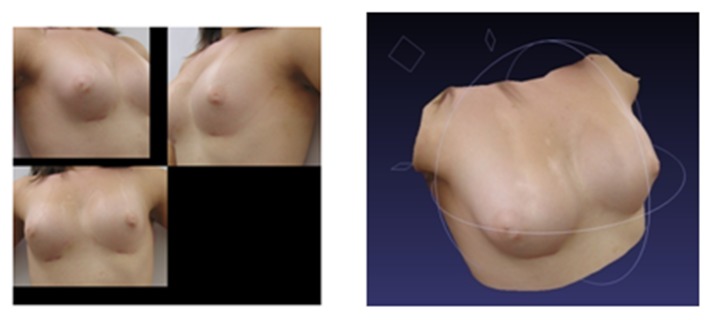
Overlapping scanner views (left) and the resulting surface scan generated by the commercial scanner software (right).

The laser scans are considered for the purposes of this study to be the criteria index, since they incorporate true depth information of the surface. To validate the algorithm, surface scans of the patient were taken at the same time as the photographs used for the 3D reconstruction. Figure 6 shows one example of the laser scanning procedure.

To construct an overlay of the resulting 3D reconstruction and the corresponding ground truth, the reconstructed breasts were aligned to the laser scan surface using surface-matching techniques in Amira 5.3.3 software (Visage Imaging GmbH, Berlin, Germany). The surface matching of the laser scan and the 3D reconstruction is required in order to bring meshes from different sources into the same coordinate system. During this surface-matching step, the shape of the breasts was preserved so as not to bias the results.

As a quantitative metric, the surface-to-surface distance between the overlaid reconstructed surface and the baseline scan was computed for each point of the reconstructed surface.

The quality control evaluation was based on a single ambulatory follow-up outpatient visit and had no implication in therapy planning for the patients. The current photographic 2D documentation was completed by a 3D camera scan to allow quality control of the efficiency of the Web-based 3D reconstruction system. Following anonymization of the data, the analysis was performed in a blinded manner. There are no prospective intents or implications in this retrospective photographic comparison. In total, we present 11 datasets, of which 10 are postoperative.

## Results

### Qualitative Analysis of 3D Reconstructions of Patient Anatomies

**Figure 7:**
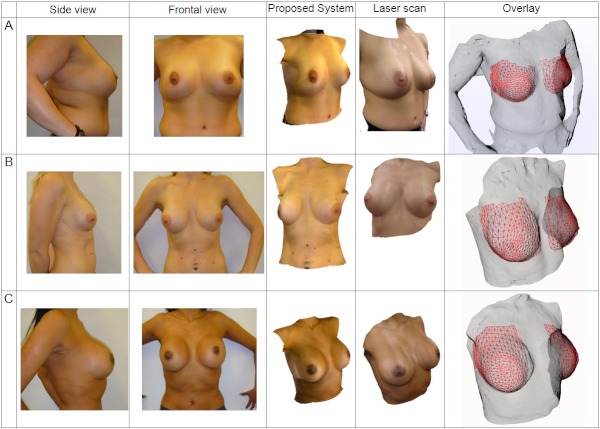
Composite figure sets showing, from left to right: patient photographs, corresponding 3-dimensional (3D) surface reconstruction, laser scan ground truth, and overlaid reconstructed surfaces. The laser scan textures were acquired in the absence of flash photography; hence, their illumination appears slightly different from that in the patient photos. The laser scan surface is shown as a transparent surface in the last column, and the 3D reconstruction is displayed as a superimposed red wire frame. The preoperative simulated images are screenshots from the Web-based interface, as seen in the Proposed System column.

Figure 7 is a composite figure that illustrates the corresponding patient photos, laser surface scan, and 3D reconstruction using the developed system. Due to space considerations, only the right lateral patient photograph is shown. Visually, the 3D reconstruction appears similar to the patient photos. We selected a variety of breast types (including frontal- and lateral-facing nipples) and patient thoraxes with different builds and skin tone to show the wide applicability of the method. The main restrictions in obtaining successful reconstructions are accurate patient poses according to the protocol and precisely positioned landmarks. From this composite figure, patients A and C show mismatches in texture brightness at the seams in the laser scan column. This is a limitation of the scanner software since, although it stitches the texture surfaces correctly, it does not compensate for the camera’s exposure value. In comparison, the system presented in this paper is better able to handle changes in illumination than the commercial scanner’s 3D reconstruction software.

To ensure that the 3D reconstruction algorithm was evaluated fairly, the breasts were cropped from the reconstructed surface in order to directly compare against the baseline laser scan, since this is the most important part of the model to present to users. The direct comparison is illustrated by the red wire-frame mesh (reconstruction) on the gray surface (laser scan) in the last column of Figure 7.

### Quantitative Analysis of 3D Reconstructions of Patient Anatomies

**Figure 8:**
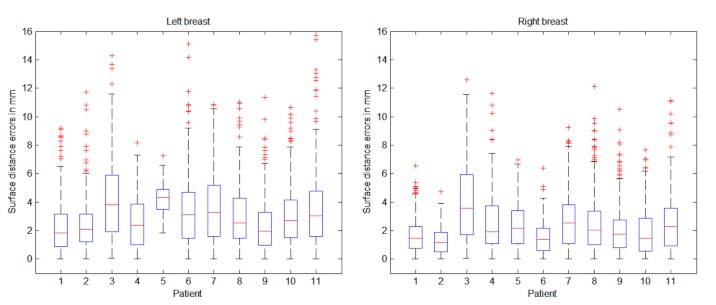
Box plots of left and right breast: 3-dimensional (3D) surface reconstructions compared with laser scans. The patients in Figure 7 correspond to cases 1, 7, and 8, respectively, in these box plots.

Visually, the overlaid reconstructions appear to correlate well with the laser scan. The results of these surface distances are shown as errors on a box plot, as displayed in Figure 8. It is important to note that this surface distance analysis compares shape only and does not account for skin texture. There is a clear correlation between the box plots for the left and right breasts, with a mean reconstruction error between 2 and 4 mm for both left and right breasts. By taking into account the large 90° angle between the frontal and lateral photographs used in the reconstruction, the observed maximum surface error appears small, and is less than the motion artifacts caused by breathing excursions and small changes due to patient repositioning between scans.

A recent paper [30] highlighted the possibility that arm positioning might affect the accuracy of breast shape reconstruction using 3D laser scanning technologies. The authors suggested investigating the effect on the shape of reconstructed breasts of arms at the sides, akimbo, and akimbo with maximal force. In relation to the accuracy attained by the proposed consultation system, our experience with scanning patients in akimbo position shows only a small craniocaudal motion of the breast as compared with arms at the sides, and very small changes to the shape of the breast. In this regard, we believe that the possible artifacts introduced by akimbo positioning are negligible compared with the reported accuracy of the developed tool. A dedicated study is, however, necessary to understand and model these aspects.

It should be noted that the aim of the system is not to compete against the very accurate 3D laser scanner technologies, but to propose a Web-based patient-specific tool to aid surgeons with the consultation process and patients with their preoperative choice.

### Quantitative and Qualitative Analysis of Biomechanical Simulations

To further control the quality of the proposed system as a breast implant consultation tool, simulations of postoperative surgery are predicted from the patients’ preoperative photos along with the knowledge of the implants chosen for the surgery. The predictions were validated using laser scans of postoperative patients. Routine photographic documentation was taken according to common surgical practice. None of these patients were presented preoperatively with a 3D reconstruction or simulation, and no clinical therapeutic decision was based on these data.

**Figure 9:**
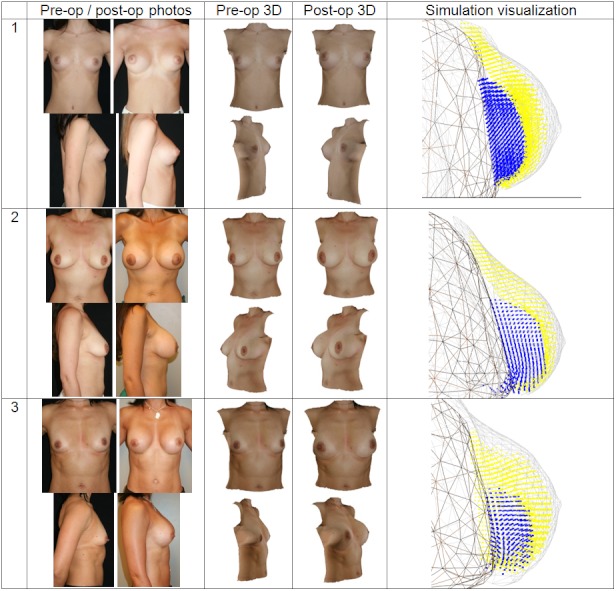
Composite figure showing pre- and postoperative photos, pre- and postoperative 3-dimensional (3D) reconstruction, and implant simulation surface renderings from the simulation visualization. The reconstructed and simulated surfaces were computed from the preoperative photos and hence show similarities in the textures. The pre- and postoperative simulated images are screenshots from the Web-based interface (middle columns).

**Figure 10:**
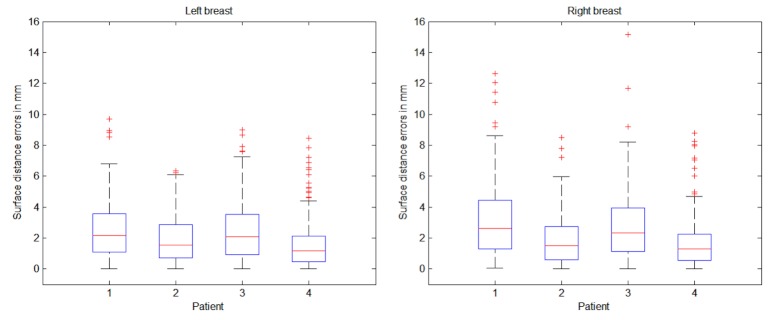
Postoperative simulation results predicted from preoperative images compared with postoperative laser scans.

Qualitative results are shown in Figure 9 and quantitative results are presented in Figure 10. Visually there is great variation in breast textures and sizes, which illustrates the applicability of the 3D reconstruction and simulation algorithms. The comparison of reconstructed and simulated breasts against postoperative laser scans was calculated in the same manner as in Figure 8. Just as in Figure 8, correlations between the left and right breast are apparent in Figure 10. The levels of mean errors in Figure 10 are similar to those for the reconstructions from postoperative photos in Figure 8. Since there are only 4 cases at this time for postoperative simulation, there are not enough grounds to draw strong conclusions. Nevertheless, the preliminary results indicate that the assumptions in the simulation do not dominate the overall errors. This could be because the reconstruction uses only 3 images to generate a full 3D surface and the simulation is physics based with material properties and uses knowledge of the patient-specific implants.

## Discussion

We present a tool developed for a 3D Web-based, patient-specific consultation in the clinical scenario of breast augmentation. The main finding of this study are that the current state of development allows for the creation of a responsive and effective Web-enabled 3D consultation tool for breast augmentation surgery based on 3D image reconstruction of 2D pictures, even with highly complex and time-consuming computation, by off-loading it to a dedicated high-performance data center.

The efficient combination of advanced technologies, based on analysis and understanding of human anatomy and physiology, will allow for the development of further Web-based reconstruction and predictive interfaces at different scales of the human body.

This consultation tool exemplifies the potential of combining advancements in the core areas of computer science and biomedical engineering along with the evolving areas of progress in Web technologies. We are confident that future developments based on a multidisciplinary approach will further pave the way toward personalized Web-enabled medicine.

### Perspectives

This technology has potential for other medical applications, such as reconstructive surgery of facial malformations, aesthetic facial and anti-aging procedures, or preoperative volume evaluations in breast reduction surgery. These areas can be targeted by modeling the different anatomical and physiological processes.

For the future development of the presented system, optimization plans include improving texturing of the reconstruction from the patient’s photographs, user retargeting of geometry [31], handling of complex breast shapes (congenital malformations, tubular breasts), and reconstructive scenarios (eg, breast cancer), as well as enhancing the Web2.0 interaction platforms between patients and surgeons.

## Abbreviations

2D: 2-dimensional
3D: 3-dimensional
TEM: tissue elastic model
